# Photoprotective Effects of Selected Amino Acids on Naproxen Photodegradation in Aqueous Media

**DOI:** 10.3390/ph13060135

**Published:** 2020-06-26

**Authors:** Kohei Kawabata, Momoka Kanoh, Mayu Okazaki, Rina Maeda, Satomi Mori, Shiori Akimoto, Masanori Inagaki, Hiroyuki Nishi

**Affiliations:** 1 Faculty of Pharmacy, Yasuda Women’s University, 6-13-1 Yasuhigashi, Asaminami-ku, Hiroshima 731-0153, Japan; 15141113@st.yasuda-u.ac.jp (M.K.); 15141110@st.yasuda-u.ac.jp (M.O.); 15141239@st.yasuda-u.ac.jp (R.M.); 15141243@st.yasuda-u.ac.jp (S.M.); inagaki@yasuda-u.ac.jp (M.I.); nishi-h@yasuda-u.ac.jp (H.N.); 2 Graduate School of Biomedical and Health Sciences, Hiroshima University, 1-2-3 Kasumi, Minami-ku, Hiroshima 734-8553, Japan; akimotos@hiroshima-u.ac.jp

**Keywords:** analytical chemistry, antioxidative activity, chromatography, HPLC, photochemistry, photodegradation, photostability, photostabilization, UV/Vis spectroscopy

## Abstract

It is important to develop a photostabilization strategy to ensure the quality of photosensitive compounds, including pharmaceuticals. This study focused on the protective effects of 20 amino acids on the photodegradation of naproxen (NX), a photosensitive pharmaceutical, to clarify the important nature of a good photostabilizer. Our previous report indicated the photodegradability of NX and the protective effects of some antioxidants on its photodegradation, therefore, this compound was used as a model compound. The degradation of NX in aqueous media during ultraviolet light (UV) irradiation and the protective effects of selected amino acids were monitored through high-performance liquid chromatography (HPLC), equipped with a reverse-phase column. Addition of cysteine, tryptophan, and tyrosine induced the significant suppression of NX photodegradation after UV irradiation for 3 h (residual amount of NX; 15.35%, 6.82%, and 15.64%, respectively). Evaluation of the antioxidative activity and UV absorption spectrum showed that cysteine suppressed NX degradation through its antioxidative ability, while tryptophan and tyrosine suppressed it through their UV filtering ability. Furthermore, three amino acids at higher concentrations (more than 100 µmol/L) showed more protective effects on NX photodegradation. For 10 mmol/L, residual amounts of NX with cysteine, tryptophan, and tyrosine were 58.51%, 69.34%, and 82.40%, respectively. These results showed the importance of both photoprotective potencies (antioxidative potency and UV filtering potency) and stability to UV irradiation for a good photostabilizer of photosensitive pharmaceuticals.

## 1. Introduction

Pharmaceuticals have been used by humans and livestock for the purpose of prevention or treatment of various diseases, all over the world. It is well-known that ultraviolet light (UV) irradiation, which is present in sunlight, provokes the loss of beneficial effects and the gain of adverse effects for photosensitive pharmaceuticals [1,2,3]. Our previous reports also indicated that photodegradation of some pharmaceuticals, containing analgesic and antiepilepsy drugs, induced ecotoxicological effects on them [4]. The energy of UV irradiation induces the excitation of chemical compounds, followed by elimination reaction, addition of the functional group, rearrangement and isomerization, and so on. Various photochemical reaction might change the physical or biological properties of photosensitive pharmaceuticals. Furthermore, UV irradiation makes a contribution to the generation of reactive oxygen species derived from the oxygen molecule, which might be a trigger of the oxidative reaction. The variety of photochemical reaction and their output as photoproducts is dependent on the variety of the chemical structure of UV-irradiated compounds and the wavelength of the UV. Additionally, UV irradiation has an effect on the content of active compounds in medicine. In the case of naproxen (NX), which is a non-steroidal anti-inflammatory drug (NSAIDs), the active compound in the tablet, its powder, and suspension was degraded by UV irradiation [5]. This report suggests that photo-degradability of pharmaceuticals is a major determinant of their quality and quantity.

To overcome the photostability problem, many efforts were made to develop photostabilization strategies in recent years. A number of reports indicate that the encapsulation is the most used approach, followed by the addition of antioxidants and solar filters [6]. In the encapsulation strategy, cyclodextrin and its modified form are major photoprotective carriers. However, in some cases, encapsulation is not able to stabilize photosensitive compounds, due to the difficulty of inclusion [7]. The report indicates that various photoprotective methods are needed for the photostabilization of various pharmaceuticals. Our previous reports showed that selected antioxidants, such as ascorbic acid derivatives and some polyphenols (quercetin, catechin, and curcumin), are protective for the photodegradation of NX, and both the antioxidative potency and the photostability of antioxidants are needed for an efficient photostabilizer [8]. Further study focused on researching the good photostabilizer is essential for the protection of photosensitive pharmaceuticals, especially those that are not protected by encapsulation.

In this study, the protective effects of selected amino acids on NX photodegradation were evaluated. NX is photosensitive for UV irradiation at longer wavelength and its degradation is suppressed by addition of some antioxidants, as shown in a previous report [8], therefore, NX is used as a test compound. Properties of amino acids are dependent on their functional groups. Comparison of protective effects of amino acids on NX photodegradation is informative for clarifying the nature of a good photostabilizer. To the best of our knowledge, there are few reports focused on the protective effects of amino acids on the photodegradation of pharmaceuticals. First, photoprotective effects of 20 amino acids were investigated. NX was photo-exposed with each amino acid, and the residual amount of NX was determined by a high-performance liquid chromatography (HPLC) system, equipped with a reverse-phase column. Second, the photoprotective mechanisms of the tested amino acids were evaluated by means of a test kit for the potential antioxidant (PAO test) and UV absorption spectral analysis. The aim of this research was to determine the protective potencies of the tested amino acids for NX photodegradation, and to elucidate the importance of its nature for a good photostabilizer. The results of this experiment might make it possible to protect photosensitive pharmaceuticals from degradation, based on various mechanisms.

## 2. Results and Discussion

### 2.1. Comparison of the Photoprotective Effects of Amino Acids on NX Photodegradation

The concept of photostabilization is important for the safe use of pharmaceuticals, which tended to be degradable by photo-irradiation. In this study, the photoprotective effects of selected amino acids for NX photodegradation were evaluated to clarify their effectiveness as a photostabilizer. The chemical structure of NX is shown in Figure S1.

The process of NX degradation after UV irradiation with or without amino acids is shown in Table 1, where the residual concentrations in % are summarized. Concentrations of NX and amino acids were 86.9 and 100 µmol/L, respectively. NX was degraded by UV irradiation and disappeared completely after 3 h. Residual amounts of NX after UV irradiation for 1, 2, and 3 h were 66.66% ± 6.22%, 2.08% ± 0.85%, and 0.00% ± 0.00%, respectively. The residual amounts of NX in the control sample after 3 h was 99.87% ± 1.22%. These results showed that only UV irradiation had a contribution to the NX degradation, and other factors such as hydrolysis and temperature had no effects on it. Addition of selected amino acids to the control sample also did not have any effect on the residual amounts of NX.

To clarify the protective effects of amino acids on NX photodegradation, 20 amino acids were added to the NX solution, followed by UV irradiation. Most of the tested amino acids had no effects on NX photodegradation, and the residual amounts of NX after UV irradiation, up to 3 h, with or without them being not significantly different (Table 1).

Amino acids categorized as the basic amino acid group (lysine, histidine, and arginine) and the acidic amino acid group (aspartic acid and glutamic acid) showed no protective effects on NX photodegrdation. It was reported that the efficiency of the photodegradation rate of some pharmaceuticals were changed by the increase or decrease of proton concentration in reaction solution [9,10]. Acidic and basic amino acids might react as a proton releaser or accepter in solution, but they did not inhibit NX photodegradation in this experiment. Additionally, the initial pH values of all amino acid solutions tested were between 6 and 7, indicating that 10 µL of 1 mol/L HCl added to the solution to support dissolution of amino acids, resulted in pH values of solution between 6 and 7.

Among the non-polar amino acid group, methionine significantly reduced the NX photodegradation after UV irradiation up to 2 h. Residual amount of NX was 18.50% ± 4.94%, which was significantly higher than that without amino acids (2.08% ± 0.85%). However, this protective effect was lost when the test solution was irradiated for 3 h. Methionine is known as a quencher for the singlet excited state of chemical compounds [11]. It is possible that methionine might quench some excited NX (about 20%), after UV irradiation until 2 h. The ability of methionine as a quencher was found to be weak, and NX was degraded completely after UV irradiation for 3 h.

**Figure 1:**
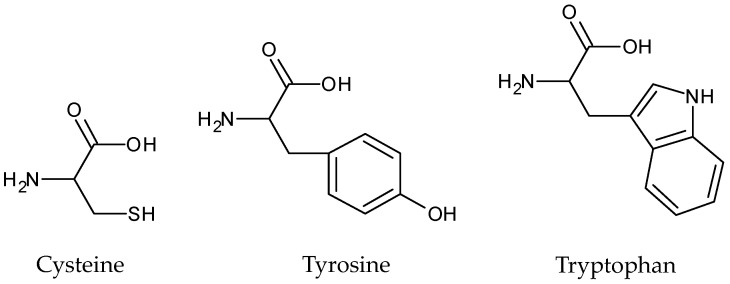
Chemical structure of cysteine, tyrosine, and tryptophan.

On the other hand, cysteine, tyrosine, and tryptophan showed protective effects on NX photodegradation after UV irradiation up to 3 h, among the non-polar amino acid group and the polar amino acid group. Their chemical structures are shown in Figure 1. Residual amounts of NX after UV irradiation were significantly higher than when other amino acids were added. Residual amounts of NX in the presence of cysteine, tyrosine, and tryptophan were 56.17% ± 2.71%, 44.97% ± 2.11%, and 26.71% ± 1.57% after UV irradiation for 2 h, and 15.35% ± 1.50%, 15.64% ± 1.96%, and 6.82% ± 1.33% for 3 h, respectively. Additionally, amounts of the NX photoproduct with cysteine, tyrosine, and tryptophan were less than that without amino acids (data not shown). These results showed that cysteine, tyrosine, and tryptophan suppressed the NX photodegradation differently from other amino acids.

### 2.2. Mechanism Elucidation of the Photoprotective Effects of Cysteine, Tyrosine, and Tryptophan

To determine the photoprotective mechanism of these amino acids, at first, the antioxidative activities of the test amino acids were examined by the PAO test. In the PAO test, only cysteine showed a reducing power. The antioxidative activity of cysteine was 297.07 ± 8.02 µmol/L. This result indicated that cysteine might act as a photostabilizer for NX photodegradation, by means of its antioxidative potency. It is reported that NX is photo-converted to its photoproducts, via the generation of radical species, followed by oxidation [12,13]. Some antioxidants, including ascorbic acid and polyphenols, suppressed NX photodegradation due to their antioxidative potencies [8]. Other studies also showed protective effects of the antioxidative agents on the photosensitive compounds [14,15]. It was speculated that cysteine might protect photosensitive pharmaceuticals from photodegradation, due to its antioxidative activity. Other report showed that, in the case of dacarbazine, cysteine suppressed the generation of its photoproduct via its potential as a superoxide scavenger, but the residual rate of dacarbazine after photo-irradiation with cysteine was the same as that without it [16]. Tyrosine and tryptophan had no antioxidative potencies in the PAO test, indicating that they showed protective effects on NX photodegradation by other natures.

Next, the UV absorption spectra of tyrosine, tryptophan, and phenylalanine were examined. The results of the UV spectral analysis of three aromatic amino acids were shown in Table 2. Tyrosine and tryptophan had the bigger molar absorption coefficients (ε) in the wavelength range around 220 nm and 280 nm, compared to phenylalanine. The absorption-maximum wavelength (λ_max_) and ε around 280 nm were as follows; phenylalanine 259 nm (238 L·mol^−1^·cm^−1^), tyrosine 278 nm (1881 L·mol^−1^·cm^−1^), and tryptophan 282 nm (6122 L·mol^−1^·cm^−1^). It was suggested that tyrosine and tryptophan might act as a UV filter, which disrupts the excitation of NX by photo-irradiation, but phenylalanine had no protective effect on the account of its low UV filtering activity. Residual amounts of NX after UV irradiation for 3 h in the presence of tyrosine and tryptophan were more than that of phenylalanine (tyrosine—15.64% ± 1.96%, tryptophan—6.82% ± 1.33%, and phenylalanine—0.00% ± 0.00%, respectively). Moreover, the protective effect of tyrosine on NX photodegradation was higher compared to tryptophan. The peak area of tryptophan after UV irradiation was lower than that of tyrosine in the HPLC analysis (data not shown), indicating that tryptophan had a tendency to be more photodegradable than tyrosine. This was in agreement with previous reports evaluated the photodegradability of some amino acids [17,18,19], and photodegradation of tryptophan and tyrosine was reported [20,21]. It was shown that both tyrosine and tryptophan acted as a UV filter, interrupting photo-irradiation for NX, and tyrosine had a more photoprotective potency compared to tryptophan, due to its higher photostability.

### 2.3. Dose Dependency of the Photoprotective Effects of Cysteine, Tyrosine, and Tryptophan

**Figure 2:**
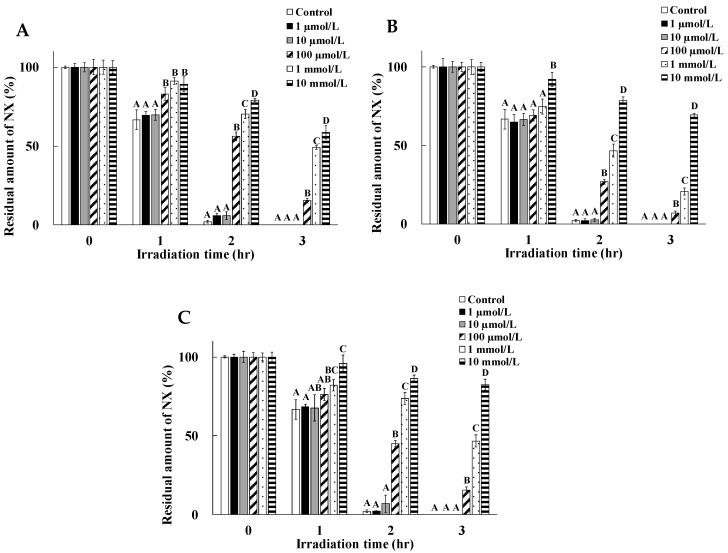
Dose dependency of the protective effects of cysteine (**A**), tryptophan (**B**), and tyrosine (**C**) on NX photodegradation. Values are mean ± S.D. (*n* = 4). Means in the same row without superscript (**A**–**D**) are significantly different (*p* < 0.05). In the absence of superscript, the means are not significantly different (*p* < 0.05). The amount of NX is shown as the residual amount of NX and the amount of NX before UV irradiation.

For the evaluation of detailed photoprotective mechanism, the dose dependency of the photostabilization effects of cysteine, tyrosine, and tryptophan were evaluated. Results are shown in Figure 2. 

It was shown that the rate of NX photodegradation was lower in the presence of cysteine, tryptophan, and tyrosine at a higher concentration. Residual amounts of NX after UV irradiation for 2 to 3 h with cysteine, tyrosine, and tryptophan were significantly higher than the control at 100 µmol/L, 1 mmol/L, and 10 mmol/L. Among three amino acids at 100 µmol/L to 10 mmol/L, tyrosine was the most effective stabilizer and the residual rate of NX in its presence was the highest, compared to cysteine and tryptophan. These results showed that the UV filtering ability is a major determinant for the photoprotective effect on NX photodegradation, compared to the antioxidative potency. Our previous reports indicated that polyphenol compounds such as quercetin and catechin showed more photostability effects on NX photodegradation, compared to ascorbic acid, on account of having more antioxidative effects [8]. It is possible that they might be a good photostabilizer as they have a polyphenol backbone, which is well-known as a UV filter. Additionally, three amino acids at 1 µmol/L and 10 µmol/L had no protective effects on NX photodegradation. It was indicated that the residual amino acids in test solutions had a contribution on the magnitude of the protective effects for NX photodegradation. Previous studies showed that the photodegradation efficiency of the irradiated compound was dependent on its initial concentration [22]. In this experiment, it was indicated that amino acids that had protective potencies at a lower concentration might tend to be degradable by photo-irradiation, compared to that at higher concentration. Based on the results shown in Figure 1, the presence of cysteine, tryptophan, and tyrosine at high concentrations, might have more protective effects on NX photodegradation, due to their own photostabilities, in addition to their antioxidative potencies or UV filtering potencies. Residual amounts of only the aromatic amino acids were examined, but that of other amino acids, such as cysteine and methionine, were not evaluated in this experiment. It was speculated that the NX photodegradation proceeded with no barrier after the disappearance of amino acids, on account of their degradation by UV irradiation.

From these results, it was important for a good photostabilizer to have antioxidative potencies, UV filtering abilities, and own photostabilities to photo-irradiation. Other chemical compounds with these potencies might be a good photostabilizer for various photosensitive pharmaceuticals. There are various reports showing the effects of UV irradiation on the persistence and biological activities of photosensitive compounds [23,24,25,26,27]. For example, the photoproduct of ametryn, which is one of herbicides, showed a higher acute ecotoxicological effect on *Vibrio fischeri* [23], and some photoproducts of cyclophosphamide and ifosfamide, which are categorized as antineoplastic drugs, might have a mutagenic potential in silico toxicity prediction [24]. On the other hand, the photoproduct of linezolid, which is one of oxazolidinone antibiotics, showed no antibacterial activities [25]. NX is also converted to its photoproducts in aqueous media and formulation by photo-irradiation [12,13], and some photoproducts showed toxic potential in ecotoxicological tests [28,29,30]. Protecting photosensitive pharmaceuticals from degradation or conversion to products is an important factor to supply safe medical care for patients.

## 3. Materials and Methods 

### 3.1. Materials

NX, Glycine, alanine, valine, leucine, isoleucine, methionine, serine, threonine, cysteine, asparagine, phenylalanine, tyrosine, tryptophan, lysine, histidine, aspartic acid, glutamic acid, methanol, ethanol, hydrochloric acid (HCl), and acetic acid were purchased from Fujifilm Wako Pure Chemical Corporation (Osaka, Japan). Proline, glutamine, and arginine were purchased from the Tokyo Chemical Industry Corporation (Tokyo, Japan). All amino acids used in this experiment were of DL-form. Milli-Q water (18.2 mΩ/cm) was prepared by using a Milli-Q water purification system (Merck, Darmstadt, Germany).

### 3.2. Preparation of the Test Solution

NX (10 mg) was initially dissolved in methanol (1 mL), and this solution was diluted with Milli-Q water to make a concentration of 10 mg/L (86.9 µmol/L). A volume of 9 mL of the diluted solution in a glass vial was used for the UV irradiation experiment. Additionally, selected amino acids were dissolved in 50% methanol to make a concentration of 50 mmol/L. When these did not dissolve easily, 10 µL of 1 mol/L HCl was added but the pH was not changed in comparison to pre-addition levels. In both conditions, the pH values of the test solutions were 6–7. In the photostabilization experiment, a test solution (9 mL) was prepared using a methanol solution of NX (43.4 mmol/L), a 50% methanol solution of amino acids (50 mmol/L) and Milli-Q water, to make a concentration of 86.9 µmol/L of NX and 100 µmol/L of each amino acid. Additionally, cysteine, tyrosine, and tryptophan solutions of 5 mol/L, 500 mmol/L, 500 µmol/L, and 50 µmol/L were prepared to evaluate the dose dependency of these amino acids. Each solution was added to the NX solution to achieve concentrations of cysteine, tyrosine, and tryptophan of 10 mmol/L, 1 mmol/L, 10 µmol/L, and 1 µmol/L.

### 3.3. UV Irradiation Experiment

UV irradiation was carried out using a light cabinet equipped with a 20 W FL20S BLB black light lamp (Toshiba, Tokyo, Japan). The most abundant wavelength of the emission light from this lamp was 365 nm, and its irradiation intensity value was 500 µW/cm^2^/s. The irradiation intensity was measured by a digital radiometer with a 365 nm sensor (UVX-36, UVP, Upland, CA, USA). The irradiation time was up to 3 h at 20 ℃. Water depth was 3.5 cm, and distance from the light source was about 15 cm. Control samples were also prepared for the same condition but covered with an aluminum foil to interrupt UV irradiation. All experiments were carried out in tetraplicates.

### 3.4. Evaluation of the Residual Amount of NX

The degradation of NX was monitored with a high-performance liquid chromatography (HPLC) system, which was composed of an LC-20 AD pump with an analytical column (Shim-pack VP-ODS, 5 µm, 4.6 × 150 mm), a SPD-20A UV detector, a CTO-20A column oven, and a C-R8A chromate-integrator (Shimadzu Corporation, Kyoto, Japan). Separation type of the HPLC system was reverse-phase chromatography. The column was kept at 40 ℃. A mixture of methanol and acetic acid (50% methanol containing 0.1% acetic acid, *v*/*v*) was used as an isocratic mobile phase at a flow rate of 1.0 mL/min. A mobile phase was prepared as follows; methanol (500 mL) and water (500 mL) were mixed, and acetic acid was added to make its concentration at 0.1% (*v*/*v*), followed by the shaking and the degas, using a sonicator and an aspirator. Detection wavelength was 254 nm. A volume of 20 µL of irradiated sample solutions were injected into an HPLC system. Retention times of NX and its main photoproduct were 22.2 min and 18.4 min. Amount of NX evaluated by HPLC are shown as % of initial compound before UV irradiation.

### 3.5. Evaluation of Antioxidative Activities

The antioxidative activities of selected amino acids were evaluated by means of the PAO test (Nikken SEIL Corporation, Shizuoka, Japan). This assay evaluated the Cu^+^ level derived from the reduction of Cu^2+^, induced by the antioxidative activities using the spectrophotometer. From these results, the antioxidative potencies of the tested samples were calculated as copper-reducing power (µmol/L). The tested amino acids were dissolved in 50% methanol to make a concentration at 100 µmol/L for the PAO test. All experiments were carried out in triplicates.

### 3.6. UV Spectral Analysis

Tested aromatic amino acids (phenylalanine, tyrosine, and tryptophan) were dissolved in ethanol at the final concentration of 100 µmol/L to 1 mmol/L. When there was difficulty in dissolving these acids, 10 µL of 0.1 mol/L HCl was added. UV absorption spectra were recorded with a V-670 UV/Vis spectrophotometer (JASCO, Tokyo, Japan), interfaced to a PC for data processing. The absorption-maximum wavelength (λ_max_, nm) of each amino acid was obtained from these results. The molar absorption coefficients (ε, L·mol^−1^·cm^−1^) were calculated from the absorption of λ_max_.

### 3.7. Statistical Analysis

Data are expressed as mean ± standard deviation (S.D.). The homogeneity of variance was established using a one-way ANOVA. Statistical significance was estimated by Tukey’s test. The threshold for assessing significance was *p* < 0.05 (vs. control).

## 4. Conclusions

From the results of the photostabilization experiments herein, cysteine, tyrosine, and tryptophan had protective effects on the photodegradation of the studied pharmaceutical. Cysteine suppressed NX photodegradation through antioxidative activity, and tyrosine and tryptophan suppressed it by UV filtering activity. Furthermore, dose dependencies of their protective effects were determined. However, in this experiment, the residual amount of the amino acids after UV irradiation, were not examined sufficiently. It is important for a good photostabilizer to have protective potencies and to be stable for photo-irradiation. Further research including investigation of other substances that have better protective potencies, and additional evaluation, are required to develop the stabilization strategy for photosensitive compounds.

## Figures and Tables

**Table 1:** Photoprotective effects of 20 amino acids on the degradation of naproxen (NX) in an aqueous media induced by UV irradiation.

Amino Acids	Categorization of Amino Acids	Residual Amounts of NX after UV Irradiation (%)								
		1 h			2 h			3 h		
-	-	66.66	±	6.22 ^A^	2.08	±	0.85 ^A^	0.00	±	0.00 ^A^
Glycine	Nonpolar	67.86	±	3.45 ^A^	4.81	±	2.50 ^A^	0.00	±	0.00 ^A^
Alanine		67.36	±	4.86 ^A^	3.99	±	1.53 ^A^	0.00	±	0.00 ^A^
Valine		70.02	±	6.69 ^A,B^	2.38	±	0.35 ^A^	0.00	±	0.00 ^A^
Leucine		74.92	±	3.04 ^A,B^	5.26	±	1.55 ^A^	0.00	±	0.00 ^A^
Isoleucine		67.24	±	5.24 ^A^	4.23	±	0.39 ^A^	0.00	±	0.00 ^A^
Phenylalanine		69.83	±	4.16 ^A,B^	5.78	±	3.02 ^A^	0.00	±	0.00 ^A^
Cysteine		82.99	±	4.03 ^B^	56.17	±	2.71 ^B^	15.35	±	1.50 ^B^
Tryptophan		69.09	±	3.61 ^A,B^	26.71	±	1.57 ^C^	6.82	±	1.33 ^C^
Proline		67.40	±	3.31 ^A^	3.25	±	1.58 ^A^	0.00	±	0.00 ^A^
Methionine		70.57	±	3.31 ^A,B^	18.50	±	4.94 ^D^	0.00	±	0.00 ^A^
Serine	Polar	69.53	±	6.88 ^A,B^	3.83	±	0.87 ^A^	0.00	±	0.00 ^A^
Threonine		72.27	±	3.05 ^A,B^	5.40	±	2.60 ^A^	0.00	±	0.00 ^A^
Tyrosine		76.14	±	3.93 ^A,B^	44.97	±	2.11 ^E^	15.64	±	1.96 ^D^
Asparagine		70.53	±	4.27 ^A,B^	1.90	±	0.61 ^A^	0.00	±	0.00 ^A^
Glutamine		73.00	±	4.47 ^A,B^	6.28	±	0.95 ^A^	0.00	±	0.00 ^A^
Lysine	Basic	67.14	±	3.38 ^A^	2.26	±	1.12 ^A^	0.00	±	0.00 ^A^
Histidine		63.52	±	2.09 ^A^	2.04	±	1.00 ^A^	0.00	±	0.00 ^A^
Arginine		69.13	±	2.70 ^A,B^	1.36	±	0.56 ^A^	0.00	±	0.00 ^A^
Aspartic acid	Acidic	72.36	±	6.58 ^A,B^	2.31	±	0.49 ^A^	0.00	±	0.00 ^A^
Glutamic acid		71.01	±	6.72 ^A,B^	1.75	±	0.32 ^A^	0.00	±	0.00 ^A^

Values are mean ± S.D. (*n* = 4). ^A–E^ indicate the identity in Tukey’s test. Means in the same row without superscript are significantly different (*p* < 0.05). In the absence of superscript, the means are not significantly different (*p* < 0.05). The amount of NX is shown as the residual amount of NX and the amount of NX before UV irradiation.

**Table 2:** Absorption-maximum wavelength (λ_max_, nm) and molar absorbance coefficient (ε, L·mol^−1^·cm^−1^) analyzed using UV/Vis spectrophotometer.

Amino Acids	Absorption-maximum Wavelength (λ_max_) and Molar Absorbance Coefficient (ε)
Phenylalanine	214 nm (2562 L·mol^−1^·cm^−1^), 259 nm (238 L·mol^−1^·cm^−1^)
Tryptophan	219 nm (28,954 L·mol^−1^·cm^−1^), 282 nm (6122 L·mol^−1^·cm^−1^)
Tyrosine	228 nm (12,216 L·mol^−1^·cm^−1^), 278 nm (1881 L·mol^−1^·cm^−1^)

## Supplementary Materials

The following are available online at https://www.mdpi.com/1424-8247/13/6/135/s1. Figure S1: The chemical structure of NX.
