# Supplementary figures and images for: Photoprotective Effects of Selected Amino Acids on Naproxen Photodegradation in Aqueous Media
Source: Pharmaceuticals (Basel). 2020 Jun 26;13(6):135. doi: 10.3390/ph13060135 (PMC7345999; doi:10.3390/ph13060135)

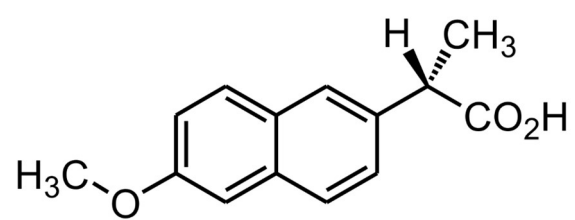

**Figure S1.** Chemical structure of NX

Supplement: Supplementary file 1 [file pharmaceuticals-13-00135-s001.pdf]
